# Skin and soft tissue infection suspiciously caused by *Klebsiella* *pneumoniae* in an aquaculture worker: A case report

**DOI:** 10.3892/mi.2024.158

**Published:** 2024-04-23

**Authors:** Caipeng Xie, Na Li, Yan Chen, Yongtao Liang, Libing Huang, Xiaoyang Xie, Dongming Wang, Haitao Wang, Guanjun Huang

**Affiliations:** 1 Department of Clinical Pharmacy, Central People's Hospital of Zhanjiang, Zhanjiang, Guangdong 524033, P.R. China; 2 Department of Rheumatology and Immunology, Central People's Hospital of Zhanjiang, Zhanjiang, Guangdong 524033, P.R. China; 3 Department of Hematology, Central People's Hospital of Zhanjiang, Zhanjiang, Guangdong 524033, P.R. China; 4 Department of Emergency Medicine, Central People's Hospital of Zhanjiang, Zhanjiang, Guangdong 524033, P.R. China; 5 Department of Clinical Laboratory, Central People's Hospital of Zhanjiang, Zhanjiang, Guangdong 524033, P.R. China

**Keywords:** aquaculture, *Klebsiella pneumoniae*, skin, soft tissue, infection

## Abstract

Skin and soft tissue infections (SSTIs), which are mainly caused by Gram-positive cocci existing on the skin surface, are more common than those caused by Gram-negative bacteria; however, the role of Gram-negative bacteria as emerging pathogens in SSTIs cannot to be ignored. *Klebsiella pneumoniae* is an opportunistic pathogenic bacterium that mainly inhabits the respiratory and intestinal tracts of humans and animals, as well as the environment, including aquaculture farms. This bacterium can cause multiple infections in humans and animals. The present study reports the case of a SSTI which was suspected to be caused by *Klebsiella pneumoniae* in a 74-year-old farmer with venous thrombosis. The patient had exposed his four bare limbs to the farmed shrimps and shrimp pond routinely. Pustule and skin ulcers were observed on both the legs of the patient. After receiving anti-infection therapy, the SSTI was almost completely resolved on day 9 and the patient was then discharged.

## Introduction

*Klebsiella pneumoniae (K. pneumoniae)* is widely distributed in nature and also colonizes the human gut and respiratory tract. It is divided into the opportunistic, hypervirulent and multi-drug resistant groups (1). It has been reported that aquaculture farms and farmed species also contain organism *K. pneumoniae* (2,3). The opportunistic strains cause respiratory tract, urinary system or blood stream infections, while hypervirulent ones can result in liver abscesses and endophthalmitis (1). The strains have also been reported to cause skin and soft tissue infections (SSTIs), including diabetic foot ulcers (4), cellulitis (5) and necrotizing fasciitis (6).

The present study describes the case of a patient with SSTI, which was suspected to have been caused by *K. pneumoniae* infection. The patient was an aquaculture worker with venous thrombosis.

## Case report

A 74-year-old male patient presented to the Department of Rheumatology and Immunology of the Central People's Hospital of Zhanjiang, Zhanjiang, China, with a complaint of redness and swelling in both lower extremities. Purulent discharge drained from the wounds scattered on the legs and some wounds were scabbing. Multiple forming scabs were observed in the upper extremities. There was no complaint of fever. He denied having a history of diabetes. The patient, an aquaculture worker, worked in the shrimp pond routinely. Of note, 6 months prior, after completing his work, he had noted pitting edema over his lower legs with an elevated skin temperature, but no obvious tenderness. The edema was relieved when he lay flat, but became aggravated following activities. Open weeping or crusted sores had developed in all four limbs simultaneously. However, the patient continued to work at the shrimp pond with bare legs and arms. He had sought medical advice in the outpatient department of the hospital 1 month prior. At that time, laboratory tests revealed a high-sensitivity C-reactive protein (hs-CRP) level of 10.65 mg/l, a white blood cell count of 12.3x10^9^/l and a neutrophil count of 90.81%; the patient had then been treated with Cefuroxime (1 g, i.v. drip, bid) for almost 1 week.

Notably, the patient had felt pain and swelling in his left ankle 2 years prior, which was before the current presentation. Gradually, the swelling and pain involved his right ankle and both knees. The symptoms were repetitive and could be relieved by anodyne.

Upon admission to the authors' institution, the patient had a temperature of 36.7˚C, a respiratory rate of 20 breaths/min, a heart rate of 90 beats/min and a blood pressure of 101/72 mmHg. The electrocardiogram assessment was normal. On day 2, laboratory tests revealed a white blood cell count of 13.2x10^9^/l, a neutrophil count of 92.4%, a lymphocyte count of 4.3%, a hs-CRP level of 200.08 mg/l and a markedly increased CD64 index of 23.91. Procalcitonin levels were within the normal range (0-0.5 ng/ml). Urine glucose was negative, while the random blood glucose level was 5.39 mmol/l. A color Doppler ultrasound (data not shown) revealed a small amount of effusion, synovial thickening and degenerative changes in the ankles, as well as arthritis in the knee joints. Immune system diseases were not considered, as no abnormalities in the examination results were found in antinuclear antibody, anti double stranded DNA antibody IgG, as well as the markers of vasculitis and rheumatoid arthritis. The diagnosis of SSTI was thus established.

**Figure 1:**
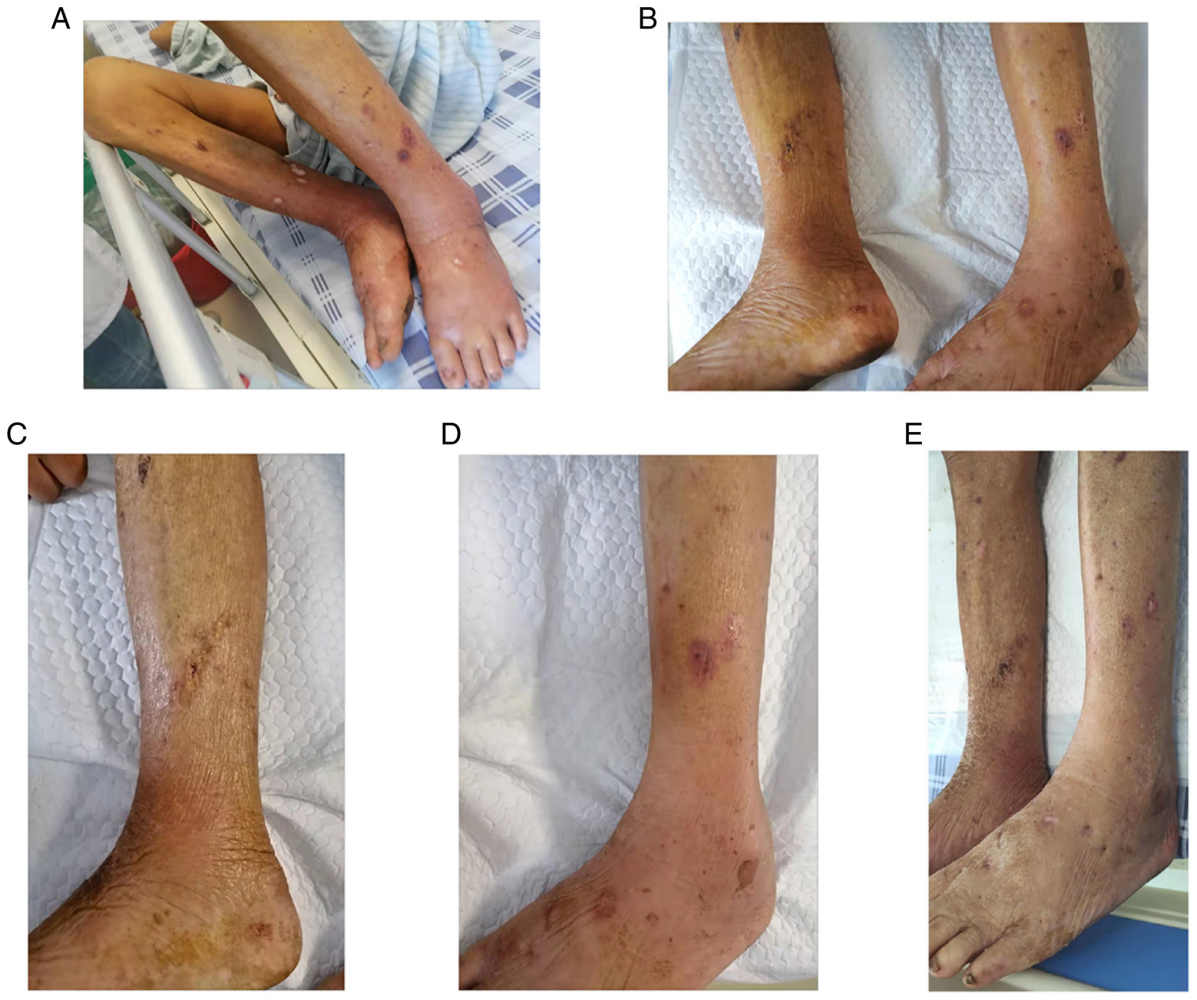
The status of the patient's lower extremities. (A) Upon admission; (B) day 4, (C) and (D) right and left legs, respectively; (E) day 8.

**Figure 2:**
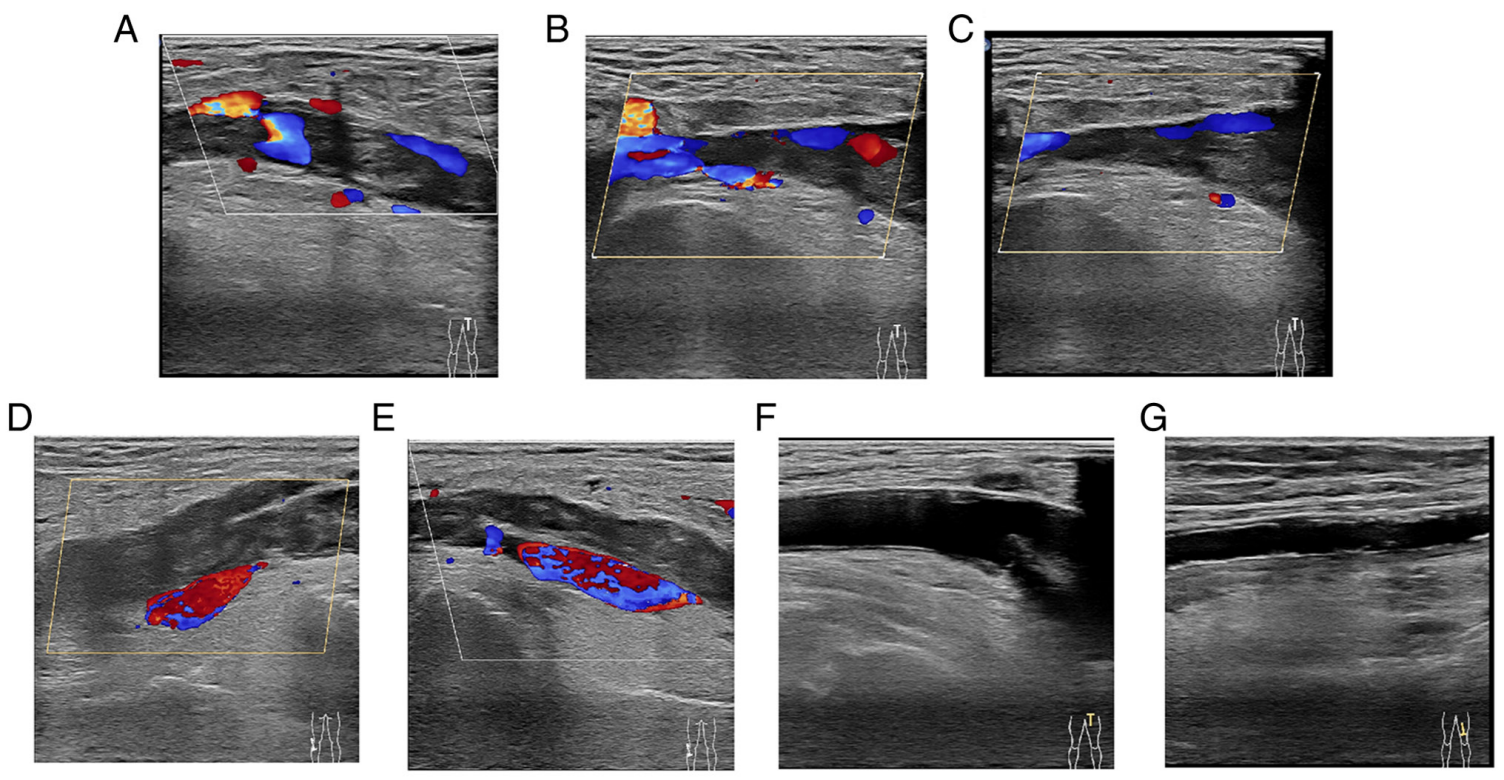
Color Doppler ultrasound image of the left lower extremity. Color Doppler ultrasound revealed incomplete thrombosis of the left common femoral vein and superficial femoral vein (A-C), and complete thrombosis of the left popliteal vein (D and E), as well as atherosclerosis of the left lower extremity (F and G).

Following the diagnosis of SSTI, the wounds of the patient were washed with normal saline and the secretion fluid from the ulceration (Fig. 1A) was sampled for pathogenic microbe culture upon admission. The patient was then commenced on treatment with Cefamandole Nafate (1 g i.v. drip, q8h) and a wet compress of ethacridine lactate solution was applied to the wounds on day 1 since admission. On day 3, the pus culture grew *Pneumoniae* subspecies (also named Friedlander bacillus), one sub-species of *K. pneumoniae*, which was identified using a fully automatic microbial identification system (VITEK 2 Compact system, bioMérieux France). The treatment was successful in relieving the redness and swelling. The wounds were found to have no fluid leakage. On day 4, the hs-CRP level was downregulated to 135.78 mg/l. The skin of the patient recovered (Fig. 1B-D). Pathogenic microbes in the skin sample could not be detected on day 6. However, the edema of the left leg worsened again. The patient had remained lying in bed in the past few days and the edema re-occurred as soon as he got up. A color Doppler ultrasound subsequently confirmed deep vein thrombosis and atherosclerosis in the left low extremities (Fig. 2), which explained the recurrent edema. The related indices of coagulation function, apart from fibrinogen (5.17 g/l) were in the normal range (2-4 g/l) and treatment was initiated with the anticoagulant, Rivaroxaban. The anti-infection therapy was maintained. With the redness and swelling exhibiting a marked reduction, the SSTI was almost completely resolved on day 9. The patient was then discharged.

## Discussion

SSTI, which is mainly caused by Gram-positive cocci, such as *Staphylococcus aureus* existing on the skin surface is more common than that of Gram-negative bacteria. The role of Gram-negative bacteria as emerging pathogens in SSTIs cannot to be ignored, with isolated microorganisms mainly comprising of *Pseudomonas aeruginosa, K. pneumoniae, Escherichia coli* and *Enterobacter cloacae* (7,8).

In the case presented herein, pustule and skin ulcers were observed on both the legs of the patient. Forming scars were also scattered over both upper extremities. The patient had exposed his four bare limbs to the shrimp pond for a long period of time. The impaired skin indicated a possible skin infection. A blood examination further confirmed the infection. The pathogen examination indicated was *K. pneumoniae*. The secondary infection of the ulcer surface on the limbs was considered. As has been reported, skin ulcers, disrupting the natural defense of the skin, as well as causing scar formation and poor blood perfusion, lead to a decrease in local immunity and thus render the ulcer surface an ideal site for bacterial proliferation and invasion (8). With development, skin ulcers become aggravated or even systemic infections can occur (8). Furthermore, chronic post-thrombotic syndrome on the left lower extremity was characterized by extremity pain, edema, venous claudication, skin changes and skin ulceration, and inflammatory responses impaired the skin barrier functions (9-12). As regards the patient in the present study, the swollen and fragile skin and soft tissue of the limbs, followed by frequent exposure to and stimulation by the shrimp pond water environment for a long period of time, may have led to further damage to the skin barrier.

On the one hand, *K. pneumoniae* is a conditional pathogen, originally colonizing in human mucous membranes, including the gastrointestinal tract and the oropharynx. However, when transported to distant sites or when it invades other tissue, it can cause infection. Skin ulcers increase the opportunistic infection of the bacterial colonization. As regards the patient described herein, perhaps *K. pneumoniae* invaded the distantly susceptible skin and induced the secondary infection. It is also possible for *K. pneumoniae* to spread to the patient's open wounds, causing secondary infection via other contact methods.

On the other hand, as the patient had continued the work even though he began to show symptoms, the susceptible skin became further damaged and the vulnerability to the infection by microorganisms in the aquaculture environments increased. Samples from water, sediment and farmed species from fish and shrimp farms include organisms that have been shown to be associated with human opportunistic infections, such as *Escherichia coli*, *Pseudomonas putida* and *K. pneumoniae* (13); however, the incidence and prevalence of the skin damage caused by *K. pneumoniae* and other bacteria within aquaculture environments as the source remain unknown. Of note, *Escherichia coli, Pseudomonas putida* and *K. pneumoniae* are widely present throughout the natural environment as conditional pathogenic bacteria. *Escherichia coli* and *K. pneumoniae*, belonging to the Enterobacteriaceae family, have been reported to be involved in diabetic foot ulcers, necrotizing fasciitis, burn wounds or other necrotizing soft tissue infections (14-18). As they sometimes may develop into extended spectrum β-lactamase-, carbapenem- and multiple drug-resistant strains, it is better to cultivate bacteria on the surface of ulcers, test drug sensitivity and select appropriate antibiotics (14-18). *Pseudomonas putida*, also a Gram-negative bacteria, is an uncommon cause of SSTIs. It is often associated with trauma or an immunocompromised state with malnutrition, immobility and peripheral vascular disease as risk factors (19-22). Gentamicin, amikacin, levofloxacin, ceftazidime, ciprofloxacin, carbapenems and other antibiotics can be used for the clinical treatment of *Pseudomonas putida* (19-22). When related infections caused by the aforementioned Gram-negative bacteria occur, the clinical characteristics include an increased white blood cell count, elevated CRP levels or sometime symptoms of fever. The aquaculture environment is the reservoir of certain bacteria, such as *K. pneumoniae*, which can be further transferred by the aquaculture supply chains (2,23,24). The prevalence of *K. pneumoniae* in aquaculture farms may be due to high stocking density, organic matter levels and poor quality of the aquatic environment in the intensive systems (25). *K. pneumoniae* may be associated with both fish and human infections, and has pathogenic significance for humans, in addition to SSTIs, also causing food-borne diseases, meningitis, urinary tract infections; thus, the potential risk in aquaculture and public health should not be underestimated (3,24). It has also been reported that multi-drug resistant *K. pneumoniae* is present in aquaculture environments, such as shrimp farms and imported shrimp (2,3,26,27). Therefore, based on the cultured strain *K. pneumoniae* in pus, the present study suggests that *K. pneumoniae* infection in the patient was perhaps related to the long-term exposure of the skin ulcers to the shrimp pond. It can be deduced that long-term exposure to an aquaculture environment in which *K. pneumoniae* is prevalent, is likely to increase the risk of *K. pneumoniae* infection; for example, *Vibrio vulnificus* infection can be caused by exposure to the marine environment (28). As regards the role of the aquaculture environment as a potential source of *K. pneumoniae* infection, aquaculture-associated bacteria transferred between animals and humans via aquaculture supply chains may require further attention. Larger numbers of samples and clinical cases need to be analyzed in order to prove that human opportunistic SSTI can be caused by aquaculture-associated *K. pneumoniae*.

In the case presented herein, infection by other pathogens was not detected. However, it could not be completely ruled out that the cultured *K. pneumoniae* was the contaminating bacteria. The possibility of a Gram-positive co-infection can also not be ruled out. Following the treatment of the patient with a wet compress of ethacridine lactate solution and Cefamandole nafate empirically for the SSTI, the patient finally exhibited a good response against the *K. pneumoniae* infection identified.

The response indicated the sensitivity of the strain isolated. As Cephalosporin is a time-dependent antibiotic, the exposure lasting time of pathogenic bacteria to the antibiotic in the body is closely related to the antibacterial effect. The longer the concentration of antibiotic is maintained over the minimum inhibitory concentration, the better the antibacterial effect will be. The poorer effect of Cefuroxime (1 g i.v.drip, bid) used in the outpatient department before may partly have been due to its usage (bid) or insufficient treatment course.

In conclusion, the present study reported a suspected case of *K. pneumoniae* induced-SSTI accompanied by venous thrombosis-induced edema in an aquaculture worker.

## Data Availability

The datasets used and/or analyzed during the current study are available from the corresponding author on reasonable request.
